# Nontraumatic coma in the pediatric intensive care unit: etiology, clinical characteristicsand outcome

**DOI:** 10.3906/sag-2004-330

**Published:** 2021-02-26

**Authors:** Muhterem DUYU, Zeynep KARAKAYA, Selin YILDIZ

**Affiliations:** 1 Department of Pediatrics, Pediatric Intensive Care Unit, İstanbul Medeniyet University, Göztepe Training and Research Hospital, İstanbul Turkey; 2 Department of Pediatrics, İstanbul Medeniyet University, Göztepe Training and Research Hospital, İstanbul Turkey

**Keywords:** Coma, nontraumatic, children, pediatric critical care, etiology, outcome

## Abstract

**Background/aim:**

The purpose of this study was to evaluate the etiology, clinical characteristics, and outcome of nontraumatic coma (NTC) among children admitted to a pediatric intensive care unit (PICU).

**Materials and methods:**

A total of 159 children with NTC were included in the study. The modified Glasgow coma scale (GCS) was used to assess consciousness. Patients were classified with regard to etiology. For each patient, demographic and clinical characteristics, survival and degree of disability at PICU discharge were recorded.

**Results:**

Median age was 55 months (IQR: 17.0 - 109.0). The most common cause of NTC was neuroinfection (31.4%) followed by toxic-metabolic conditions (25.8%) and epileptic disorder (15.1%). There was no significant relationship between the level of encephalopathy at admission and NTC etiology. A total of 13 patients died (8.2%). Among the survivors, 61.6% were discharged without any neurologic deficit, 2.8% had severe neurologic disability, and 3.4% were in a vegetative state. Complete neurological recovery was significantly more common in patients with toxic metabolic disease, whereas neurological deficits were more frequent in patients with tuberculous meningo-encephalitis (P = 0.01 and P = 0.04, respectively). Higher pediatric risk of mortality III (PRISM III) score at PICU admission (Odds ratio: 1.51, 95% CI: 1.19 - 1.92; P < 0.001) was the only variable that was independently associated with mortality. The length of stay (LOS) at hospital (Odds ratio: 0.73, 95% CI: 0.58-0.91; P = 0.006) was associated with improved odds of survival.

**Conclusions:**

Although results obtained from this single-center study cannot be generalized to the pediatric population, the contribution to the literature in terms of the relationships between NTC etiology, and outcome can be crucial for clinical decision-making. We report neuroinfection as the most common cause of NTC, and the only factor that was closely associated with mortality was PRISM III score. Length of hospital stay was inversely correlated to patient mortality.

## 1. Introduction

Coma is a state of altered level of consciousness in which there is a loss of both wakefulness and awareness of the self and the environment. It is a very serious condition that necessitates immediate medical decision making upon arrival at the pediatric emergency department or pediatric intensive care unit (PICU) [1]. The most common scoring system used for assessment of consciousness is the Glasgow coma scale (GCS), and a modified version of the GCS is used in pediatric patients [2,3]. Pediatric coma is generally defined as having a GCS of < 12 for at least 6 h [4]. Coma, without a history of a traumatic event, is an accompanying feature of many different conditions, including infectious, metabolic-toxic, and epileptic etiologies. These conditions, or the coma resulting from them, can be fatal if they are not swiftly identified and sufficiently treated [5,6].

Usually, as a first diagnostic step, discrimination is made between traumatic and nontraumatic coma (NTC). However, in the pediatric population, the determination of the cause of NTC (and thus, its etiology) is a challenge for the physician; however, it is vital to making appropriate treatment decisions. Etiology of coma and clinical status at the time of admission are likely outcome predictors. Although structural and nonstructural causes of coma may overlap each other, discrimination should be made between destructive structural brain lesion (cerebral hemisphere or brain stem) and global acute physiological derangement of brain function by performing case-specific investigations in order to be able to manage patients appropriately [7]. 

Most epidemiological studies about coma in children have focused on traumatic coma [8,9]. The majority of NTC studies in children have investigated the prognostic analysis of specific etiologies (e.g. super-refractory convulsive status epilepticus, posterior reversible encephalopathy, virus associated encephalitis etc.) [10,11,12]. There are only a few studies in which NTC has been evaluated in terms of severity, etiology, and prognosis [13,14]. In this study, our aim was to determine the etiology and clinical characteristics of patients and the factors that were associated with mortality and degree of disability in pediatric patients with NTC who were admitted to a tertiary-hospital PICU.

## 2. Materials and methods

This is a single-center, retrospective, and descriptive study approved by İstanbul Medeniyet University Clinical Research Ethics Committee (clinical study registration: 2019/0265). The study was conducted on patients aged 1 month to 17 years who were admitted to our tertiary PICU between March 2015 and November 2019. The modified GCS scoring system was used [3]. The GCS of patients and its relationship with prognosis and etiology were assessed by dividing the study population into three groups depending on the level of consciousness at admission: the first group was comprised of those with a GCS score of 10-12 (mild encephalopathy), the second group had moderate encephalopathy (GCS: 7-9), and the third group had severe encephalopathy (GCS: 3-6) [14,15,16].

All patients who had a GCS of 12 or lower for at least 6 h at admission were included, given that they had normal neurological development and did not have any history of trauma. Exclusion criteria were as follows: (I) having a history of trauma, (II) having a disease of the central nervous system (CNS) or a systemic disease affecting the CNS, (III) having a modified GCS higher than 12, (IV) having a modified GCS score lower than 12 for less than 6 h.

The etiology of coma was determined on the basis of history, clinical examination, neurological imaging, and relevant laboratory investigations. Specific investigations, such as cerebrospinal fluid analysis, liver function tests, arterial blood gas analysis, electroencephalogram, and neuroimaging studies (computerized tomography, CT or magnetic resonance imaging, MRI) were performed whenever necessary. 

For each patient, the following data were collected: demographic and clinical characteristics, admission pediatric risk of mortality III (PRISM III) score and predictive death rate (%PDR), clinical presentation (seizure, vomiting, fever, headache, focal neurological deficit, neck rigidity, personality changes, speech disorder, abnormal pupillary reflex [ *absence of pupillary light reflex and/or unequal pupillary light reflex* ], diplopia, ataxia, opisthotonus), modified GCS score at admission, mechanical ventilator requirement, length of stay (LOS) at the PICU and hospital, survival, and degree of disability at PICU discharge. The etiology of NTC was classified as follows: (I) neuroinfection, (ii) toxic-metabolic, (iii) epileptic, (iv) autoimmune disease, (v) stroke / vascular pathology, (vi) cns tumour, (vii) hypoxic ischemic encephalopathy, (viii) hypertensive encephalopathy, and (ix) unknown etiology.

We evaluated degree of disability at PICU discharge using the pediatric cerebral performance category (PCPC) scales [17]. Scores on the PCPC run from 1 to 5, with the following definitions: no symptoms (1: normal); alert and able to interact at age-appropriate level but school-age children may not have appropriate levels for age even though they attend regular school classroom, possibility of mild neurologic deficit (2: mild disability); sufficient cerebral function for age-appropriate independent activities of daily life, school-age child attending special education classroom and/or having learning deficit (3: moderate disability); dependent on others for daily activity because of impaired brain function (4: severe disability); unaware, even if awake in appearance, does not interact with environment, cerebral unresponsiveness and no evidence of cortex function, possibility of some reflexive response, spontaneous eye-opening, and sleep-wake cycles (5: vegetative state).

### 2.1. Statistical analysis

The data obtained from the study were evaluated via the IBM SPSS (Version 15.0, SPSSInc., Chicago, IL, USA) statistical package program. Normality of distribution was tested (Shapiro-Wilk and Kolmogorov-Smirnov) and normal distribution assumptions for the respective tests were not met in any analysis. The frequency, percentage (%), median, interquartile range (IQR) were used for the depiction of descriptive data. Categorical data were compared using Chi-square tests. The Mann-Whitney U test was used to compare continuous variables. Univariate logistic regression analysis and multivariate logistic regression analysis were used to assess which factors were effective on mortality (dependent variable). Variables which were found to be associated with mortality in the univariate analysis were determined as independent variables. Statistical significance level was accepted as P < 0.05.

## 3. Results

### 3.1. Clinical characteristics and etiology 

During the study period, a total of 1541 patients were admitted to our PICU. Among these, 27.4% (n = 422) were diagnosed with coma. The number of patients excluded according to each exclusion criterion are as follows: 151 patients due to traumatic coma, 46 patients due to having > 12 modified GCS, 34 patients because they had primary CNS disease or a systemic disease with known CNS involvement, and 32 patients due to having GCS < 12 for a duration shorter than 6 h. Finally, 159 encephalopathic children with a diagnosis of NTC were included in the study. The incidence of NTC meeting these criteria was 10.3% of PICU admissions. Among these, 81 cases (50.9%) were male and median age was 55 months (IQR: 17.0 - 109.0). The subjects included 33 children (20.8%) aged from 1 month to 1 year, 54 children (34.0%) aged from 1 year to 5 years and the greatest proportion of cases with 72 children (45.2%) were aged > 5 years. The median PRISM III score and %PDR at admission were 20.0 (IQR: 16.0 - 26.0) and 24.5 (IQR: 34.5 - 64.6) respectively. The most common clinical presentation was seizure (52.2%), followed by vomiting (41.5%) and fever (40.9%). Children with NTC had a median GCS score of 9 (IQR: 7 - 11) at admission, almost half of them had mild encephalopathy (49.0%) followed by moderate encephalopathy (36.5%) and severe encephalopathy (14.5%). Most patients needed mechanical ventilator support (67.9%). The median LOS at PICU was 6 days (IQR: 3 - 14), median LOS at hospital was 16 days (IQR: 6 - 27). Patients’ demographic and clinical characteristics are detailed in Table 1.

**Table 1:** Demographic, clinical parameters, and outcome of children with nontraumatic coma.

Patients characteristics	Value
Sex, n (%)	
Male	81 (50.9)
Female	78 (49.1)
Age (month), median (IQR)	55.0 (17.0-109.0)
Age group, n (%)	
1 month-1 year	33 (20.8)
1-5 years	54 (34.0)
> 5 years	72 (45.2)
PRISM score on admission, median (IQR)	20.0 (16.0-26.0)
PDR (%) on admission, median (IQR)	24.5 (34.5-64.6)
Clinical presentation, n (%)	
Seizure	83 (52.2)
Vomiting	66 (41.5)
Fever	65 (40.9)
Headache	30 (18.9)
Focal neurological deficit	17 (10.7)
Neck rigidity	11 (6.9)
Personality changes	11 (6.9)
Speech disorder	8 (5.0)
Abnormal pupillary reflex	8 (5.0)
Diplopia	7 (4.4)
Ataxia	7 (4.4)
Opisthotonus	5 (3.1)
Glasgow coma scale (GCS) on admission, median (IQR)	9 (7–11)
Encephalopathy level on admission, n (%)	
Mild (GCS 10-12)	78 (49.0)
Moderate (GCS 7-9)	58 (36.5)
Severe (GCS 3-6)	23 (14.5)
Need of ventilator support, n (%)	
Required	108 (67.9)
Not required	51 (32.1)
LOS at PICU (days), median (IQR)	6 (3-14)
LOS at hospital (days), median (IQR)	15 (6-27)
Survival, n (%)	
Alive	146 (91.8)
Dead	13 (8.2)
Degree of disability at PICU discharge, n (%)	
Normal (w/o any disability)	90 (61.6)
Mild	14 (9.6)
Moderate	33 (22.6)
Severe	4 (2.8)
Vegetative	5 (3.4)

The most common cause of NTC was neuroinfection (n = 50, 31.4%). Among these patients, the most common infectious etiology was viral encephalitis (n = 24, 15.0%) followed by acute bacterial meningitis (n = 13, 8.1%). The second and third most common etiologies were toxic-metabolic causes (n = 41, 25.8%) and epileptic causes (n = 24, 15.1%), respectively. Other causes included autoimmune diseases (9.4%), stroke/vascular pathologies (6.3%) and CNS tumors (3.8%). The remaining 8 patients (5%) had ‘unknown etiology’. Detailed etiological profile has been summarized in Table 2. 

**Table 2:** Etiological classification of patients with nontraumatic coma.

Etiology	Frequency n (%)
Neuroinfection	50 (31.4)
Viral encephalitis	24 (15.0)
Acute bacterial meningitis	13 (8.1)
Virus-associated encephalitis	7 (4.4)
Tuberculosis meningo-encephalitis	2 (1.3)
Other	4 (2.6)
Toxic-metabolic	41 (25.8)
Neurometabolic diseases	14 (8.8)
Pharmaceutical poisoning	9 (5.7)
Diabetic ketoacidosis	8 (5.0)
Hepatic encephalopathy	6 (3.8)
Carbon monoxide poisoning	4 (2.5)
Epileptic	24 (15.1)
Febrile status epilepticus	13 (8.2)
Afebrile status epilepticus	11 (6.9)
Autoimmune disease	15 (9.4)
Autoimmune encephalitis	5 (3.1)
Acute disseminated encephalomyletitis	3 (1.9)
Autoimmune lymphoproliferative disease	2 (1.3)
Thrombotic thrombocytopenic purpura	1 (0.6)
Hashimato thyroiditis	1 (0.6)
Takayasu’s arteritis	1 (0.6)
Kawasaki disease	1 (0.6)
Haemolytic uremic syndrome	1 (0.6)
Stroke/Vascular pathology	10 (6.3)
Arteriovenous malformation	5 (3.3)
Sinus vein thrombosis	1 (0.6)
Moya moya disease	1 (0.6)
Ischemic infarct	1 (0.6)
Aneurysm rupture	1 (0.6)
Hemorrhagic disease of the newborn	1 (0.6)
Central nervous system tumor	6 (3.8)
Ependymoma	3 (1.9)
Astrocytoma	2 (1.3)
Arachnoid cyst	1 (0.6)
Hypoxic ischemic encephalopathy	3 (1.9)
Hypertensive encephalopathy	2 (1.3)
Unknown etiology	8 (5.0)
Total	159 (100.0)

There was no difference between boys and girls in terms of etiological distribution. Neuroinfection was significantly more common in the 1 month–1 year age group (33.3%) (P = 0.04). Clinical presentations were analyzed in relation to NTC etiologies, and it was found that seizure was most common in the epileptic etiology group. Further characterization of patients in this regard showed that fever, vomiting, and neck rigidity were most frequent in the infectious group; focal neurological signs were most common in the stroke/vascular pathologies group, while diplopia and ataxia were more common in the CNS tumors group (P < 0.05 for all). There was no significant relation between level of encephalopathy and the different etiologies of NTC (P = 0.09). 

### 3.2. Outcome 

Overall, 146 patients (91.8%) survived and 13 patients died (8.2%). Detailed etiologic distribution of dead children is shown in Table 3. Among the 146 survivors, 61.6% were discharged from PICU without any neurological deficit, 9.6% had mild disability, 22.6% had moderate disability, 2.8% had severe disability, and 3.4% were in a vegetative state (Table 1).

**Table 3:** Detailed analysis evaluating the effect of etiological groups on mortality and morbidity of children with nontraumatic coma.

		Outcome			Disability level					
Etiology, n (%)	Total	Alive	Dead	P value	Normal	Mild	Medium	Severe	Vegetative	P value
Neuroinfection	50 (100.0)	46 (92.0)	4 (8.0)	0.96	25 (54.3)	7 (15.2)	11 (23.9)	1 (2.2)	2 (4.3)	0.54**
Viral encephalitis	24 (100.0)	23 (95.8)	1 (4.2)	0.39	14 (60.8)	3 (13.1)	5 (21.7)	1 (4.4)	0 (0.0)	0.54**
Enterovirus	9 (100.0)	9 (100.0)	0 (0.0)		8 (88.9)	0 (0.0)	1 (11.1)	0 (0.0)	0 (0.0)	
Herpes simplex virus	6 (100.0)	6 (100.0)	0 (0.0)		2 (33.3)	3(50.0)	0 (0.0)	1 (16.7)	0 (0.0)	
Cytomegalovirus	3 (100.0)	3 (100.0)	0 (0.0)		2 (66.7)	0 (0.0)	1 (33.3)	0 (0.0)	0 (0.0)	
Adenovirus	2 (100.0)	2 (100.0)	0 (0.0)		1 (50.0)	0 (0.0)	1 (50.0)	0 (0.0)	0 (0.0)	
Ebstein-Barr virus	1 (100.0)	1 (100.0)	0 (0.0)		0 (0.0)	0 (0.0)	1 (100.0)	0 (0.0)	0 (0.0)	
Varicella zoster virus	1 (100.0)	1 (100.0)	0 (0.0)		1 (1000)	0 (0.0)	0 (0.0)	0 (0.0)	0 (0.0)	
Human herpes virus 7	1 (100.0)	1 (100.0)	0 (0.0)		0 (0.0)	0 (0.0)	1 (100.0)	0 (0.0)	0 (0.0)	
Rabies virus	1 (100.0)	0 (0.0)	1 (100.0)		0 (0.0)	0 (0.0)	0 (0.0)	0 (0.0)	0 (0.0)	
Acute bacterial meningitis*	13 (100.0)	11 (84.6)	2 (15.4)	0.33	6 (54.5)	3 (27.3)	1 (9.1)	0 (0.0)	1 (9.1)	0.09**
Neisseria meningitidis	6 (100.0)	4 (66.7)	2 (33.3)		2 (50.0)	2 (50.0)	0 (0.0)	0 (0.0)	0 (0.0)	
Streptococcus pneumoniae	3 (100.0)	3 (100.0)	0 (0.0)		2 (66.7)	0 (0.0)	1 (33.3)	0 (0.0)	0 (0.0)	
Haemophilus influenzae	2 (100.0)	2 (100.0)	0 (0.0)		2 (100.0)	0 (0.0)	0 (0.0)	0 (0.0)	0 (0.0)	
Escherichia coli	1 (100.0)	1 (100.0)	0 (0.0)		0 (0.0)	1 (100.0)	0 (0.0)	0 (0.0)	0 (0.0)	
Staphylococcus aureus	1 (100.0)	1 (100.0)	0 (0.0)		0 (0.0)	0 (0.0)	0 (0.0)	0 (0.0)	1 (100.0)	
Virus-associated encephalitis	7 (100.0)	6 (85.7)	1 (14.3)	0.55	3 (50.0)	0 (0.0)	3 (50.0)	0 (0.0)	0 (0.0)	0.37**
Influenza virus	5 (100.0)	4 (80.0)	1 (20.0)		2 (50.0)	0 (0.0)	2 (50.0)	0 (0.0)	0 (0.0)	
Rotavirus	1 (100.0)	1 (100.0)	0 (0.0)		0 (0.0)	0 (0.0)	1 (100.0)	0 (0.0)	0 (0.0)	
Parainfluenza virus	1 (100.0)	1 (100.0)	0 (0.0)		1 (100.0)	0 (0.0)	0 (0.0)	0 (0.0)	0 (0.0)	
Tuberculosis meningo-encephalitis	2 (100.0)	2 (100.0)	0 (0.0)	1.00	0 (0.0)	0 (0.0)	1 (50.0)	0 (0.0)	1 (50.0)	0.01**
Other	4 (100.0)	4 (100.0)	0 (0.0)	1.00	2 (50.0)	1 (25.0)	1 (25.0)	0 (0.0)	0 (0.0)	0.16**
Hydatid Cyst	1 (100.0)	1 (100.0)	0 (0.0)		1 (100.0)	0 (0.0)	0 (0.0)	0 (0.0)	0 (0.0)	
Actinomyces viscosus	1 (100.0)	1 (100.0)	0 (0.0)		0 (0.0)	0 (0.0)	1 (100.0)	0 (0.0)	0 (0.0)	
Clostridium tetani	2 (100.0)	2 (100.0)	0 (0.0)		1 (50.0	1 (50.0)	0 (0.0)	0 (0.0)	0 (0.0)	
Toxic- metabolic	41 (100.0)	39 (95.1)	2 (4.9)	0.38	26 (66.7)	3 (7.7)	7 (17.9)	2 (5.1)	1 (2.6)	0.04**
Neuromethabolic disease	14 (100.0)	13 (92.9)	1 (7.1)	0.53	2 (15.4)	2 (15.4)	6 (46.2)	2 (15.4)	1 (7.7)	NA
Urea cycle defect	2 (100.0)	2 (100.0)	0 (0.0)		1 (50.0)	0 (0.0)	1 (50.0)	0 (0.0)	0 (0.0)	
Fatty acid oxidation defect	1 (100.0)	1 (100.0)	0 (0.0)		0 (0.0)	0 (0.0)	1 (100.0)	0 (0.0)	0 (0.0)	
aFructose 1-6 diphosphatase	1 (100.0)	1 (100.0)	0 (0.0)		1 (100.0)	0 (0.0)	0 (0.0)	0 (0.0)	0 (0.0)	
Neurotransmitter defect	1 (100.0)	0 (0.0)	1 (100.0)		0 (0.0)	0 (0.0)	0 (0.0)	0 (0.0)	0 (0.0)	
Lipid storage disease	2 (100.0)	2 (100.0)	0 (0.0)		0 (0.0)	0 (0.0)	1 (50.0)	1 (50.0)	0 (0.0)	
Mitochondrial cytopathy	2 (100.0)	2 (100.0)	0 (0.0)		0 (0.0)	0 (0.0)	0 (0.0)	1 (50.0)	1 (50.0)	
Unspecified	5 (100.0)	5 (100.0)	0 (0.0)		0 (0.0)	2 (40.0)	3 (60.0)	0 (0.0)	0 (0.0)	
Pharmaceutical poisoning	9 (100.0)	9 (100.0)	0 (0.0)	1.00	9 (100.0)	0 (0.0)	0 (0.0)	0 (0.0)	0 (0.0)	NA
Diabetic ketoacidosis	8 (100.0)	8 (100.0)	0 (0.0)	1.00	7(87.5)	0 (0.0)	1 (12.5)	0 (0.0)	0 (0.0)	NA
Hepatic encephalopathy*	6 (100.0)	5 (83.3)	1 (16.7)		5 (100.0)	0 (0.0)	0 (0.0)	0 (0.0)	0 (0.0)	NA
Carbon monoxide poisoning	4 (100.0)	4 (100.0)	0 (0.0)	1.00	3 (75.0)	1 (25.0)	0 (0.0)	0 (0.0)	0 (0.0)	NA
Epileptic	24 (100.0)	24 (100.0)	0 (0.0)	1.00	22 (91.7)	1 (4.2)	0 (0.0)	1 (4.2)	0 (0.0)	0.27**
Febrile status epilepticus	13 (100.0)	13 (100.0)	0 (0.0)	1.00	13 (100.0)	0 (0.0)	0 (0.0)	0 (0.0)	0 (0.0)	0.06**
Afebrile status epilepticus	11 (100.0)	11 (100.0)	0 (0.0)	1.00	9 (81.8)	1 (9.1)	0 (0.0)	1 (9.1)	0 (0.0)	0.22**
Autoimmune diseases	15 (100.0)	14 (93.3)	1 (6.7)	0.82	6 (42.9)	1 (7.1)	7 (50.0)	0 (0.0)	0 (0.0)	0.85**
Autoimmune encephalitis	5 (100.0)	4 (80.0)	1 (20.0)		1 (25.0)	1 (25.0)	2 (50.0)	0 (0.0)	0 (0.0)	
bADEM	3 (100.0)	3 (100.0)	0 (0.0)		1 (33.3)	0 (0.0)	2 (66.7)	0 (0.0)	0 (0.0)	
cALPD	2 (100.0)	2 (100.0)	0 (0.0)		1 (50.0)	0 (0.0)	1 (50.0)	0 (0.0)	0 (0.0)	
dTTP	1 (100.0)	1 (100.0)	0 (0.0)		0 (0.0)	0 (0.0)	1 (100.0)	0 (0.0)	0 (0.0)	
Hashimato thyroiditis	1 (100.0)	1 (100.0)	0 (0.0)		1 (100.0)	0 (0.0)	0 (0.0)	0 (0.0)	0 (0.0)	
Takayasu’s arteritis	1 (100.0)	1 (100.0)	0 (0.0)		0 (0.0)	0 (0.0)	1 (100.0)	0 (0.0)	0 (0.0)	
Kawasaki disease	1 (100.0)	1 (100.0)	0 (0.0)		1 (100.0)	0 (0.0)	0 (0.0)	0 (0.0)	0 (0.0)	
Hemolytic uremic syndrome	1 (100.0)	1 (100.0)	0 (0.0)		1 (100.0)	0 (0.0)	0 (0.0)	0 (0.0)	0 (0.0)	
Stroke/Vascular pathology	10 (100.0)	7 (70.0)	3 (30.0)	0.02	1 (14.3)	2 (28.6)	2 (28.6)	0 (0.0)	2 (28.6)	0.29**
Arteriovenous malformation	5 (100.0)	2 (40.0)	3 (60.0)		0 (0.0)	1 (50.0)	0 (0.0)	1 (50.0)	0 (0.0)	
Sinus vein thrombosis	1 (100.0)	1 (100.0)	0 (0.0)		0 (0.0)	1 (100.0)	0 (0.0)	0 (0.0)	0 (0.0)	
Moya moya disease	1 (100.0)	1 (100.0)	0 (0.0)		0 (0.0)	0 (0.0)	1 (100.0)	0 (0.0)	0 (0.0)	
Ischemic infarct	1 (100.0)	1 (100.0)	0 (0.0)		0 (0.0)	0 (0.0)	1 (100.0)	0 (0.0)	0 (0.0)	
Aneurysm rupture*	1 (100.0)	1 (100.0)	0 (0.0)		1 (100.0)	0 (0.0)	0 (0.0)	0 (0.0)	0 (0.0)	
Hemorrhagic disease of the newborn	1 (100.0)	1 (100.0)	0 (0.0)		0 (0.0)	0 (0.0)	0 (0.0)	0 (0.0)	1 (100.0)	
Central nervous system tumor	6 (100.0)	6 (100.0)	0 (0.0)	1.00	1 (16.7)	0 (0.0)	5 (83.3)	0 (0.0)	0 (0.0)	0.05**
Ependymoma	3 (100.0)	3 (100.0)	0 (0.0)		0 (0.0)	0 (0.0)	3 (100.0)	0 (0.0)	0 (0.0)	
Astrocytoma	2 (100.0)	2 (100.0)	0 (0.0)		0 (0.0)	0 (0.0)	2 (100.0)	0 (0.0)	0 (0.0)	
Arachnoid cyst	1 (100.0)	1 (100.0)	0 (0.0)		1 (100.0)	0 (0.0)	0 (0.0)	0 (0.0)	0 (0.0)	
Hypoxic ischemic encephalopathy	3 (100.0)	0 (0.0)	3 (100.0)	1.00	0 (0.0)	0 (0.0)	0 (0.0)	0 (0.0)	0 (0.0)	NA
Hypertensive encephalopathy	2 (100.0)	2 (100.0)	0 (0.0)	1.00	2 (100.0)	0 (0.0)	0 (0.0)	0 (0.0)	0 (0.0)	0.87**
Unknown etiology	8 (100.0)	8 (100.0)	0 (0.0)	1.00	7 (87.5)	0 (0.0)	1 (12.5)	0 (0.0)	0 (0.0)	0.62**
Total	159 (100.0)	146 (91.8)	13 (8.2)		90 (61.6)	14 (9.6)	33 (22.6)	4 (2.8)	5 (3.4)	

* Group referenced in logistic regression. ** P value obtained as a result of in-group Chi-square test. a Fructose 1-6 diphosphatase deficiency. b Acute disseminated encephalomyelitis. c: Autoimmune lymphoproliferative disease. d

The relationships between NTC etiology and patient outcome are presented in Table 3 and Table 4. Mortality risk was found to be 5.96-fold (95% CI: 1.33-26.62) higher in those who had stroke/vascular pathology as compared to those who did not (P = 0.02). Other etiological factors did not have a significant effect on mortality. Comparisons revealed that complete neurological recovery was significantly more common in patients with toxic metabolic disease, whereas neurological deficits were more frequent in patients with tuberculous meningo-encephalitis (P = 0.01 and P = 0.04, respectively).

**Table 4:** Univariate and multivariate logistic regression analysis for prediction of mortality.

Variables	Total	Alive	Dead	Univariate analysis		Multivariate analysis	
				Odd’s ratio (95% CI)	P value	Odd’s ratio (95% CI)	P value
Sex							
Male, n (%)	81 (100.0)	74 (91.4)	7 (8.6)	1.14 (0.36-3.54)	0.83		
Female, n (%)*	78 (100.0)	72 (92.3)	6 (7.7)				
Age (month), median (IQR)	55.0 (17.0-109.0)	55.5 (17.8-107.3)	36.0 (7.5-179.0)	1.00 (0.99-1.01)	0.77		
Age group, n (%)							
1 month- 1 year	33 (100.0)	28 (84.8)	5 (15.2)	(0.64-8.92)	0.19		
1-5 years	54 (100.0)	51 (94.4)	3 (5.6)	0.79 (0.18-3.45)	0.75		
> 5 years*	72 (100.)	67 (93.1)	5 (6.9)				
PRISM score, median (IQR)	20.0 (16.0-26.0)	19.0 (15.0-24.0)	36.0 (32.0-41.0)	1.33 (1.17-1.52)	< 0.001	1.51 (1.19-1.92)	0.001
PDR (%), median (IQR)	34.5 (18.7-64.6)	29.9 (15.7-54.6)	93.5 (86.3-97.6)	1.00 (0.99-1.01)	0.15		
Encephalopathy level on admission							
Mild (GCS 10-12), n (%)*	78 (100.0)	76 (97.4)	2 (2.6)				
Medium (GCS 7-9), n (%)	58 (100.0)	55 (94.8)	3 (5.2)	2.07 (0.33-12.82)	0.43	0.31 (0.02-5.23)	0.42
Severe (GCS 3-6), n(%)	23 (100.0)	15 (65.2)	8 (34.8)	20.27 (3.91-105.05)	<0.001	1.87 (0.09-37.73)	0.68
Need of ventilator support							
Required, n (%)*	108 (100.0)	95 (88.0)	13 (12.0)				
Not required, n (%)	51 (100.0)	51 (100.0)	0 (0.0)	0.00 (0.00-..)	0.99		
LOS in PICU (day), median (IQR)	6 (3-14)	6.0 (3.0-14.3)	5.0 (3.0-7.0)	0.95 (0.87-1.04)	0.24		
LOS in hospital (day), median (IQR)	15 (6-27)	16.0 (9.0-28.0)	5.0 (3.0-7.0)	0.82 (0.71-0.94)	0.004	0.73 (0.58-0.91)	0.006
Etiology of coma, n (%)							
Neuroinfections	50 (100.0)	46 (92.0)	4 (8.0)	0.96 (0.28-3.30)	0.96		
Toxic-metabolic	41 (100.0)	39 (95.1)	2 (4.9)	0.50 (0.11-2.35)	0.38		
Epileptic	24 (100.0)	24 (100.0)	0 (0.0)	0.00 (0.00-…)	1.00		
Autoimmune	15 (100.0)	14 (93.3)	1 (6.7)	0.79 (0.09-6.50)	0.82		
Stroke/Vascular pathology	9 (100.0)	7 (77.8)	2 (22.2)	5.96 (1.33-26.62)	0.02	1.94 (0.00-118315.40)	0.91
Central nervous system tumor	6 (100.0)	6 (100.0)	0 (0.0)	0.00 (0.00-…)	1.00		
Hypoxic ischemic encephalopathy	2 (100.0)	2 (100.0)	0 (0.0)	23558593304 (0.00-…)	1.00		
Hypertensive encephalopathy	3 (100.0)	0 (0.0)	3 (100.0)	0.00 (0.00-…)	1.00		
Unknown etiology	8 (100.0)	8 (100.0)	0 (0.0)	0.00 (0.00-…)	1.00		

* Referenced group in logistic regression. PRISM: Pediatric risk of mortality; PDR: Predicted death rate; GCS: Glasgow coma score; LOS: Length of stay; PICU: Pediatric intensive care unit; IQR: Interquartile range.

In the univariate logistic regression analysis performed to determine factors affecting mortality, it was found that the risk of death was associated with high PRISM III score at admission (Odds ratio: 1.33, 95% CI: 1.17-1.52; P < 0.001), severe encephalopathy at admission (Odds ratio: 20.27, 95% CI: 3.91-105.05; P < 0.001), and presence of stroke/vascular pathology (Odds ratio: 5.96, 95% CI: 1.33-26.62; P = 0.020) (Table 4). From all clinical presentations, only the presence of abnormal light reflex (unequal or nonreactive) was found to increase mortality risk (Odds ratio: 8.46, 95% CI: 1.76-40.60; P = 0.008). The LOS at hospital was associated with improved odds of survival (Odds ratio: 0.82, 95% CI: 0.71-0.94; P = 0.004). Sex, age, %PDR, need for ventilator support, LOS at PICU, other etiological groups, and clinical presentations of NTC were not found to be significant for the prediction of mortality.

Based on multivariate logistic regression analysis, only the presence of higher PRISM III score at PICU admission (Odds ratio: 1.51, 95% CI: 1.19-1.92; P < 0.001) was independently associated with mortality. The LOS at hospital was associated with improved odds of survival (Odds ratio: 0.73, 95% CI: 0.58-0.91; P = 0.006). Severe encephalopathy on admission, presence of stroke/vascular pathology, and presence of abnormal light reflex were not found to be significant in multivariate analysis (P = 0.68, P = 0.91, P = 0.16, respectively).

## 4. Discussion 

NTC is a significant health problem with considerable morbidity and mortality in children. Timely diagnosis and identification of pathological mechanisms and treatment may be helpful in reducing morbidity and mortality. This study reveals several important results about pediatric NTC by reporting characteristics and relationships in its presentation, cause, mortality, and shortterm neurological outcome.

According to our study, the majority of patients were in the > 5 years age group. In comparison, a study from Nigeria found that the majority of children were between 1-5 years of age (62.5%) [18]. Another study from Saudi Arabia reported higher incidence among young infants [19]. We found that there was no influence of sex on etiological distribution. However, regarding age groups in relation to etiology, neuroinfection was most common in the 1 month to 1 year age group. A study from Iran found infectious encephalopathy most commonly in children < 2 years, whereas toxic coma was significantly more frequent in the 2-6 year age group [20].

The majority of patients admitted to the PICU with NTC in the current study had mild encephalopathy. Previous studies in children have demonstrated varying frequencies of encephalopathy findings at admission. For instance, a study from Nigeria reported 79.5% of children were deeply comatose at admission [18]. We also evaluated clinical presentations in relation to NTC etiology and found that seizure was (expectedly) the most common presentation in the epileptic group, while the other relationships were as follows: fever, vomiting, and neck rigidity in infectious etiologies; focal neurological signs in stroke/vascular etiologies; diplopia and ataxia in CNS tumors. However, overall, there was no significant relationship between level of encephalopathy and the different etiologies of NTC. In a study from Japan, seizure incidence was lower in herpes simplex virus (HSV) encephalitis as compared to the other causes, whereas abnormal speech and behavior was reported to be associated with influenza related encephalitis [21].

Among the children with NTC, neuroinfectious etiology was the most common cause of encephalopathy with viral encephalitis being the most frequent diagnosis. A study from Turkey found that hypoxic ischemic encephalopathy (HIE) and neuroinfection were the most common causes of NTC [22]. Similarly, studies from other countries like Pakistan, India, United Kingdom, Nigeria, Malaysia, and Saudi Arabia also reported neuroinfection as a leading cause of NTC [4,13,19,23-25]. In the current study, among the etiological agents of viral encephalitis, we found that enterovirus and HSV were present in more than half of the children. Similar results were found previously in a study from Pakistan [23]. However, the type and pattern of neuroinfection differs with regard to the characteristics of the country. For example, cerebral malaria was predominant in Africa, while dengue fever was common in South East Asia [24,26]. In Japan, virus-associated encephalitis was reported as the most common cause of acute encephalitis with influenza and rotavirus as the leading pathogens [27]. In our study, when we evaluated patients with neuroinfection, virus-associated encephalitis was found as the 3rd most common etiology of NTC following viral encephalitis and acute bacterial meningitis. Within the etiological agents of virus-associated encephalitis, we found influenza as the most common causative agent, indicating a resemblance to the study from Japan. In our study, the most common etiology of acute bacterial meningitis was Neisseria meningitides, similar to a multicenter surveillance study from Turkey that reported the same organism as the most frequently encountered cause of acute bacterial meningitis in children [28]. It is interesting that various unusual pathological agents were identified in the study, including Rabies virus, Clostridium tetani, and Hydatid cyst. Also, a wide variety of etiologies including rare diseases were present within the current study population, indicating that our study group was diverse and heterogeneous. This was most probably caused by the fact that ours was a tertiary healthcare center with a high number of referrals from other centers.

Among the noninfectious causes of NTC, toxic-metabolic etiologies were reported rather frequently in previous studies [13,25]. Particularly in developed countries, HIE and toxic-metabolic causes are determined in a great majority of patients [29,30]. In our study, the second most common etiology of NTC was toxic-metabolic causes. A study from Iran that included 123 children reported the most common cause of NTC as toxic encephalopathy (followed by infectious encephalopathy) [20]. In a series from Nigeria, epileptic encephalopathy was reported as the second most common cause of NTC [24]. In contrast, epileptic encephalopathy was the third frequent etiology in our study. Unlike pediatric studies, infectious causes are less frequent in adult studies. In a large series including 865 adult patients, the most common etiologies of coma were toxic (poisoning) causes and stroke (intracranial hemorrhage or infarct) [31]. Stroke is rarely encountered in the pediatric age group and the diagnosis is usually delayed. It must be noted that, in the pediatric population, such rare etiologies should be considered in the differential diagnosis of coma and early neurological imaging should be performed in case of suspicion [32]. In our study, stroke/vascular pathologies were found in 6.3% of patients, and the most common underlying condition was arteriovenous malformation. 

The current study reports a mortality rate of 8.2% in pediatric patients with NTC, whereas mortality rates in the literature range between 12.0% and 36.0% [4,13,23,25]. Also, it is important to note that previous studies report higher mortality rate in NTC compared to traumatic coma [19]. An important proportion of patients (61.6%) were discharged without any disability in the current study. This is in contrast with the report of Forsyth et al. who highlighted the presence of high cognitive dysfunction in children with NTC [33]. In a study from India, 11% of children had full recovery, while a very high proportion of patients (54%) had neurological impairment [34]. 

Logistic regression analysis for the accurate prediction of mortality and short-term neurological disability revealed no influence of age and sex on mortality and neurological sequelae. In India, a higher mortality in children < 3 years was found due to higher frequency of toxic and metabolic causes, intracranial bleeding, and neuroinfection in this age group [13]. In our study, mortality risk was found to be 5.9 times higher in those who had stroke/vascular pathology as compared to those who did not. However, in multivariate analysis, this relationship was insignificant. 

We also evaluated whether clinical findings at admission were related to mortality. We concluded that, only the presence of abnormal pupillary light reflex was associated with mortality; however, this relationship was insignificant when assessed as an independent risk factor. Previous studies have reported that the absence of pupillary light reflex is associated with unfavorable neurological outcome and mortality [22]. In the present study, the absence of pupillary light reflex and unequal pupillary light reflex findings were evaluated together, which may have caused variations in results. The relationships between mortality and GCS score at admission have been exhaustively investigated. In the literature, many studies report that low GCS score is associated with mortality and unfavorable neurological outcome [15,35]; whereas, studies reporting no relationship also exist [19]. In our study, we could not find a relationship between GCS score and mortality or neurological outcome. Only high PRISM III score was associated with mortality as an independent risk factor. Also, the length of hospital stay was found to be positively related with survival rate. We think that this is due to the fact that survivors had longer stay due to the continuation of their treatment and rehabilitation process.

There are some limitations to be discussed. Firstly, the retrospective nature of the study prevented the evaluation of long-term outcomes. Even though short-term outcomes were assessed at discharge, these examinations cannot replace follow-up studies of cognitive and psychosocial functions after discharge. Thus, our results may be hampered by the possibility of cognitive worsening after PICU discharge. Furthermore, the limitations of neurological assessment in infants may also be an important problem that could have been avoided if long-term evaluations could have been performed. Finally, although appropriate statistical analyses were applied to minimize error, this study cannot be generalized to the whole pediatric population in Turkey. However, we believe our study group is an extremely good representation of NTC cases that can be seen in any center, particularly due to the high number of patient referrals that are often received from the whole country.

A better understanding about etiology and outcome in pediatric patients with NTC will be helpful in the management of such children. In the current study, the most common etiology of NTC was neuroinfection followed by toxic metabolic causes in our pediatric population. However, cause of NTC may vary according to the development level of countries, geographical location and even due to differences between regions in the same country. Therefore, for the differential diagnosis of coma, clinicians should be aware that NTC causes may differ depending on the region. Among the many clinical and epidemiological characteristics evaluated in patients with NTC, we found that only high PRISM III score was associated with mortality. Length of hospital stay was significantly longer in survivors. This may be due to the possibility that diseases of CNS may require longer treatment and rehabilitation. Prospective multicenter studies with larger groups following patients for a longer period of time will be useful for better evaluation and analysis of factors affecting neurological outcome and survival of patients. 

## Informed Consent

All participants signed an informed consent form before participating in the study.

## Ethical Statement

This is single-center, retrospective, and descriptive study approved by İstanbul Medeniyet University Clinical Research Ethics Committee (clinical study registration: 2019/0265). 

## Contributors

All authors have agreed on the final version of this manuscript. 

DM: study design, statistical analysis, drafted the manuscript, and is responsible for the overall content. 

KZ: Contributed to the writing of the manuscript and revised the manuscript for important intellectual content. 

YS: Data extraction and quality assessment.
